# Immune Response Targeting Sjögren’s Syndrome Antigen Ro52 Suppresses Tear Production in Female Mice

**DOI:** 10.3390/ijms19102935

**Published:** 2018-09-27

**Authors:** Marta Trzeciak, Harini Bagavant, Joanna Papinska, Umesh S. Deshmukh

**Affiliations:** Arthritis and Clinical Immunology Program, Oklahoma Medical Research Foundation, Oklahoma City, OK 73104, USA; Marta-Trzeciak@omrf.org (M.T.); Harini-Bagavant@omrf.org (H.B.); Joanna-Papinska@omrf.org (J.P.)

**Keywords:** Sjögren’s syndrome, Ro52, lacrimal gland, dry eye, sexual dimorphism

## Abstract

Autoantibodies reactive against Ro52 are present in 70% of Sjögren’s syndrome patients and are associated with higher disease severity. However, their role in causing aqueous deficient dry eye, a major cause for morbidity in Sjögren’s syndrome, is unclear. To investigate whether immune responses targeting Ro52 contribute towards the dry eye, male and female NZM2758 mice were immunized with recombinant Ro52. Tear production was measured by the phenol red thread test. Sera were analyzed for anti-Ro52 levels by immunoprecipitation. Lacrimal glands were evaluated for inflammatory foci and IgG deposits. Our results showed that, although all mice generated anti-Ro52 antibodies, only females developed a significant drop in tear production. None of the mice developed severe lacrimal gland inflammation, and female mice with anti-Ro52 showed higher levels of IgG deposits within their glands. Passive transfer of anti-Ro52 sera caused reduced tear production in female mice, but not in males. This study demonstrates for the first time that immune responses initiated by Ro52 induce aqueous dry eye, and this may be driven by anti-Ro52 antibodies. Furthermore, the sexual dimorphism in glandular dysfunction suggests that the lacrimal glands in females are more susceptible to autoantibody-mediated injury.

## 1. Introduction

Dry eye is one of the major complications of Sjögren’s syndrome, affecting almost 90% of patients [1]. Dry eye leads to constant eye irritation, and in some cases blurred vision. Dry eye disease in Sjögren’s syndrome is caused by reduced fluid secretion by the lacrimal gland, as well as by the evaporative loss of tear volume [2]. Multiple factors, such as autoantibodies, inflammatory cell infiltrates, and proinflammatory cytokines can potentially influence lacrimal gland function [3,4,5,6]. Autoimmune mechanisms of dry eye disease have been investigated in rodent models using immunization with crude lacrimal gland extracts [7,8,9], purified kallikrein 1b22 [10], and acinar cell microparticles [11] as antigens. While these immunizations induce lacrimal gland inflammation and dry eye disease, the literature on immune responses to these antigens in Sjögren’s syndrome patients is limited [12,13].

A characteristic feature of Sjögren’s syndrome is the presence of circulating autoantibodies, particularly those targeting the Ro/SSA antigens [14]. Autoantibodies against Ro/SSA are an important criterion for the classification of Sjögren’s syndrome patients [15,16]. The anti-Ro/SSA autoantibodies target two distinct proteins, Ro60, also known as TROVE2, and Ro52, known as TRIM21. Ro60 is an RNA binding protein and it is implicated in the removal of misfolded RNA transcripts [17]. By contrast, Ro52/TRIM21 does not bind RNA and belongs to a large family of tripartite motif-containing proteins [18]. Functionally, Ro52 is an E3 ubiquitin ligase and is involved in either the degradation or stabilization of different proteins involved in the regulation of innate immune responses [19,20,21]. In addition, Ro52 is the only known intracellular protein that binds the Fc region of IgG antibodies and hence functions as an intracellular Fc receptor [22]. In Sjögren’s syndrome patients, anti-Ro52 autoantibodies are associated with higher disease severity [23,24], but their role in the pathogenesis of dry eye disease is not known.

Previous work from our laboratory has demonstrated that immune responses against whole Ro52 or a fragment containing its coiled-coil domain reduce pilocarpine-induced salivation in mice [25,26]. The salivary gland dysfunction in this experimental model was antibody-mediated and was dependent on the activation of innate immunity. To elucidate the role of anti-Ro52 in dry eye, in this study, we immunized NZM2758 mice of both sexes with a recombinant maltose binding protein (MBP)-mouse Ro52 fusion protein (MBP-mRo52). Control mice were immunized with MBP alone. In some experiments, a high titer anti-Ro52 immune serum was passively transferred into NZM2758 male and female mice. Our data show that anti-Ro52 reduced tear production only in female mice. Antibody deposition within the lacrimal gland appears to be responsible for the induction of glandular dysfunction. Our results suggest that female lacrimal glands are more susceptible to immune-mediated injury.

## 2. Results

### 2.1. Ro52-Immunized NZM2758 Female Mice Develop Lacrimal Gland Dysfunction

**Figure 1:**
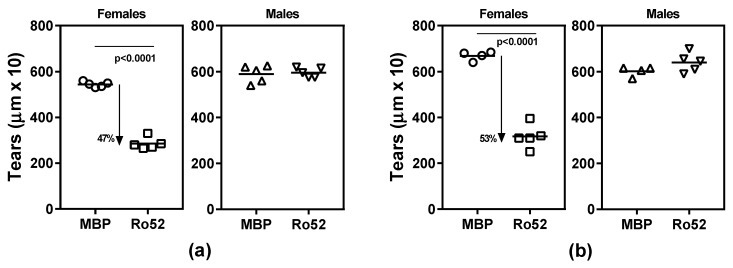
Loss of tear production in Ro52-immunized NZM2758 female mice. (**a**) Tear collection done 4 weeks post-immunization; (**b**) Tear collection done 10 weeks post-immunization in a different cohort of mice. In both panels, only female mice show a significant drop in mean tear volume.

Previous studies from our laboratory have demonstrated that, 4 weeks after immunization with Ro52 or its fragment containing the coiled-coil domain, female NZM2758 mice develop salivary gland dysfunction [25,26]. In the current study, to determine whether immune response to Ro52 affects lacrimal gland function, tear production in immunized mice was measured by using the phenol red thread test (Figure 1). Female mice immunized with recombinant Ro52 protein showed a significant drop (47%, *p* < 0.0001) in mean tear production compared to MBP-immunized control mice (Figure 1a). Interestingly, such a drop was not seen in age-matched male mice immunized with Ro52.

To determine whether lacrimal gland dysfunction in male mice was time-dependent, tear production was monitored in additional cohorts of mice, 10 weeks post-immunization (Figure 1b). Similar to the 4-week time point, only female mice showed a significant drop (53%, *p* < 0.0001) in mean tear volume. The Ro52-immunized male mice showed a modest, but a statistically insignificant increase in tear volume. Considering that most of the tear volume is contributed by the lacrimal gland, these data demonstrate for the first time that immune response to Ro52 induces lacrimal gland dysfunction.

### 2.2. Antibody Response to Ro52 Is Not Significantly Different between Female and Male Mice

**Figure 2:**
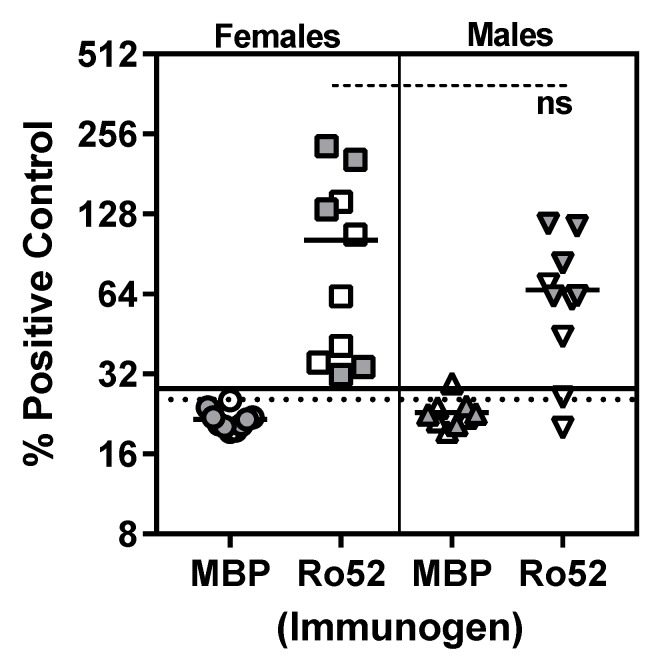
Analysis of anti-Ro52 antibody response in Ro52- and MBP-immunized mice. Sera obtained at 4 weeks (open symbols) and 10 weeks (filled symbols) post-immunization were analyzed for anti-Ro52 antibody levels by immunoprecipitating in vitro transcribed, translated and ^35^S-labeled Ro52. Data are presented as % of a positive control anti-Ro52 serum sample. The dotted line and the solid line show reactivity cut-offs for female and male mice respectively, and they were calculated as mean % positive control for MBP + 2 S.D.

To determine whether differences observed in lacrimal gland dysfunction between female and male mice were dependent on the magnitude of immune response to Ro52, antibodies to Ro52 were analyzed in an immunoprecipitation assay (Figure 2). Sera obtained at 4 and 10 weeks post-immunization were studied. Although the female mice showed a higher trend in the levels of immunoprecipitating anti-Ro52 antibody than males, this difference was statistically not significant. Further, the difference in frequency of anti-Ro52 positivity between females (10/10) and males (8/10) was statistically not significant (*p* = 0.4736, Fisher’s exact test).

### 2.3. Female Mice Show Higher Severity of Antibody Deposits within Lacrimal Glands

**Figure 3:**
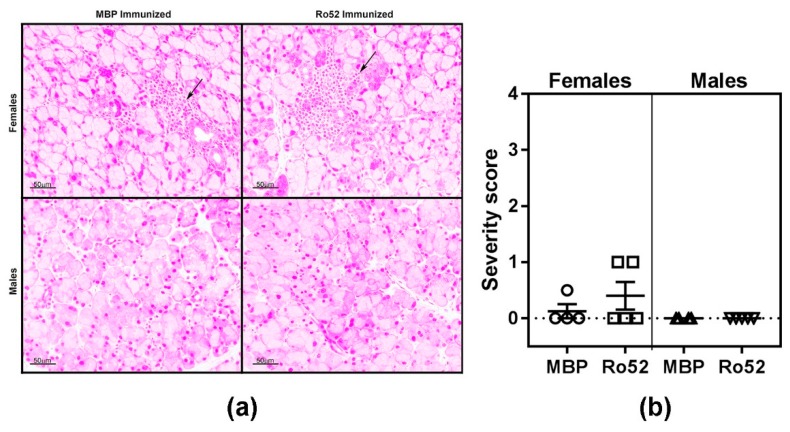
Analysis of inflammatory cell infiltration in lacrimal glands of Ro52- and MBP-immunized mice. (**a**) Representative images of hematoxylin and eosin (H&E) stained sections of lacrimal glands obtained 10–11 weeks post-immunization are shown. Arrows in the top panel show mild foci of inflammation in female mice. Such foci were not seen in male mice; (**b**) The severity of inflammation was scored on a scale of 0–4 by an observer blinded to the experimental details. All samples are from the 10 weeks post-immunization time point.

To elucidate the mechanisms responsible for lower tear production in Ro52-immunized mice, lacrimal glands obtained at the time of euthanasia were analyzed for the presence of inflammatory cell infiltrates (Figure 3a). Regardless of the immunogen (Ro52 or MBP), female mice showed the presence of mild foci of inflammation. However, there was no difference in the incidence and severity of inflammation between these 2 groups (Figure 3b). Such foci were not detected in the lacrimal glands of male mice. These data suggest that inflammatory focus formation was not the cause for inducing lacrimal gland dysfunction in Ro52-immunized mice.

**Figure 4:**
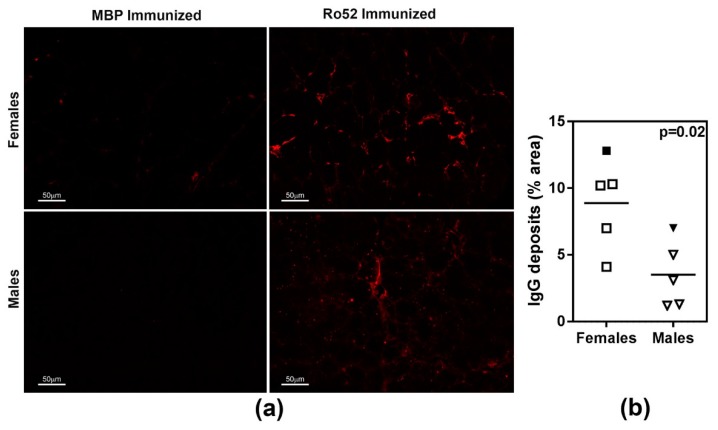
IgG deposits in lacrimal glands of female and male mice immunized with Ro52 and MBP. (**a**) The presence of IgG deposits in lacrimal glands was evaluated by direct immunofluorescence by staining lacrimal gland sections with Alexa 647-coupled goat anti-mouse IgG; (**b**) Quantitation of IgG deposits showed that Ro52-immunized female mice had significantly more IgG deposits. The nonparametric Mann-Whitney test was used to determine statistical significance and a *p* < 0.05 considered significant. Filled symbols represent the Ro52-immunized mice shown in panel (**a**).

Our previous work has demonstrated that Ro52-immunized mice develop IgG deposits in their submandibular salivary glands [25,26]. Thus, in this study, lacrimal glands were investigated for the presence of IgG deposits (Figure 4). When compared with the MBP-immunized control mice, both female and male mice in the Ro52-immunized group showed an overall higher level of IgG deposits in their lacrimal glands. However, the Ro52-immunized female mice showed considerably more IgG staining within their lacrimal glands than the Ro52-immunized male mice.

### 2.4. Passive Transfer of Anti-Ro52 Immune Sera Recapitulates Lacrimal Gland Dysfunction in Female Mice

**Figure 5:**
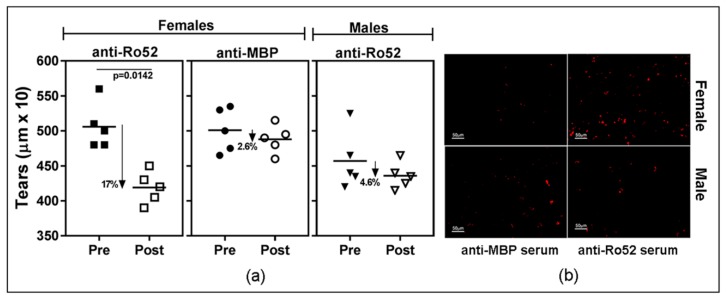
(**a**) Loss of tear production in female NZM2758 mice following passive transfer of anti-Ro52 immune sera. Alum-treated female and male mice were injected either with high titer anti-Ro52 or anti-MBP immune sera and tear production was measured after 24 h. A significant drop in mean tear volume was seen only in female mice. Paired *t*-test was used to determine statistical significance and a *p* < 0.05 considered significant; (**b**) Representative images showing rabbit IgG deposition in lacrimal glands of male and female mice following passive transfer of anti-Ro52 or anti-MBP.

To obtain further evidence on the induction of antibody-mediated lacrimal gland dysfunction, high titer anti-Ro52 and anti-MBP immune sera were passively transferred into NZM2758 that were previously treated with alum. Within 24 h after serum transfer, female mice receiving anti-Ro52 immune sera showed a significant drop (17%, *p* = 0.0142) in mean tear volume (Figure 5a, left panel). A small (2.6%), but statistically insignificant drop was seen in female mice given high titer anti-MBP sera (Figure 5a, middle panel). In an additional cohort of female mice, anti-MBP serum injection failed to cause any reduction in tear flow, while anti-Ro52 serum resulted in a 14% drop in the mean tear volume (*p* = 0.0337).

Only female mice injected with anti-Ro52 sera showed deposition of rabbit IgG in their lacrimal glands, further supporting a role for anti-Ro52 antibodies in reducing tear production (Figure 5b, top panel).

Male mice given anti-Ro52 immune sera did not have a significant drop in mean tear volume (Figure 5a, right panel), and failed to show significant IgG deposits in their lacrimal glands (Figure 5b, bottom panel). The results from the anti-Ro52 serum transfer experiments are congruent with the findings from mice actively immunized with Ro52 protein.

## 3. Discussion

In this study, by using an experimental mouse model system, we demonstrate for the first time that immune response to Ro52 is responsible for causing lacrimal gland dysfunction. Female NZM2758 mice showed reduced tear production within 4 weeks of immunization with Ro52 and this phenomenon was reproduced in mice passively transferred with high titer anti-Ro52 immune sera. Interestingly, sexual dimorphism was observed in the induction of glandular dysfunction, male mice were resistant to the antibody-mediated loss of tear production.

The role of autoantibodies in inducing exocrine gland dysfunction in Sjögren’s syndrome has mainly focused on antibodies targeting the cholinergic muscarinic receptor 3 (M3R) [27]. Considering that fluid secretion is heavily dependent on the activation of M3R by acetylcholine, it was only logical to surmise that antibodies blocking this receptor would prevent signaling and thereby suppress fluid secretion. Passive transfer of anti-M3R antibodies from human sera into mice has been reported to suppress saliva production [28]. By contrast, some of the anti-M3R antibodies have been shown to have an agonistic effect and enhance fluid secretion [29]. Whether anti-M3R autoantibodies are involved in the induction of dry eye is unclear. CD25 knockout mice that spontaneously develop multi-organ inflammation and dry eye disease were seropositive for anti-M3R autoantibodies supporting a pathogenic role for these antibodies [30]. However, their ability to experimentally suppress tear production was not evaluated. Of note are studies by Chen et al. showing that autoantibodies against the 2nd extracellular loop of M3R did not suppress either saliva or tear production [31]. In our model, we have previously demonstrated that Ro52 immunization in NZM2758 mice failed to induce circulating antibodies reactive with M3R [26], and they are therefore not the mediators of reduced saliva or tear production. Antibodies to kallikrein 13 are the only other autoantibodies described in Sjögren’s syndrome patients and implicated, albeit indirectly, in the induction of dry eye disease [32]. Mice exposed to desiccating stress developed dry eye disease and had circulating autoantibodies reactive with kallikrein 13. Passive transfer of sera from these mice to naïve mice reduced basal tear production in the recipients and induced ocular surface inflammation and damage.

Both Ro52-immunized and MBP-immunized mice showed the presence of mild foci of inflammation in the lacrimal glands. We have previously demonstrated that salivary glands from alum injected mice develop mild inflammation [33]. Thus, the mild lacrimal gland inflammation in female mice might be due to the activation of innate immunity by the adjuvant alum used for immunization. Taken together, our data suggest that the lacrimal gland dysfunction seen in Ro52-immunized mice is primarily antibody-driven. Whether these mice develop severe inflammatory foci at later time points and whether their ocular surfaces show evidence for inflammation needs to be investigated.

Sjögren’s syndrome mainly affects women, and both Sjögren’s-associated and non-Sjögren’s dry eye disease is more prevalent in women [34]. In this study, we noted that male mice were resistant to the effects of immune responses targeting Ro52. Unlike female mice, they did not significantly lose their ability to produce tears following active immunization with Ro52 or after passive transfer of high titer anti-Ro52 immune sera. These data suggest that either lacrimal glands from males are resistant to autoantibody-mediated injury or, conversely, female lacrimal glands are more susceptible to injury. This is exemplified by the finding of increased IgG deposits in female lacrimal glands. Whether these deposits are exclusively anti-Ro52 or other antibody specificities generated through epitope spreading needs to be determined. In Sjögren’s syndrome patients, anti-Ro52 antibodies often coexist with anti-Ro60 [23]. Although the Ro proteins are structurally and functionally distinct, it is possible that antibodies reactive with both proteins facilitate the pathogenic effect of anti-Ro52. In the NZM2758 mouse model, this does not seem to be the case. We have previously demonstrated that immunization of mice with the coiled-coil domain of Ro52 (amino acids 188–250) induced anti-Ro52 immune response and salivary gland dysfunction, but it did not generate antibodies to Ro60 [26]. In a preliminary investigation, we have noted that female NZM2758 mice immunized with the coiled-coil domain of Ro52 showed a 28.7% drop in tear volume when compared to the controls (*p* = 0.0003), whereas male mice immunized with this domain did not show a significant drop in tear production (3.7% drop; *p* = 0.305). Collectively, these data suggest that, in our model system, an immune response to Ro52 alone is sufficient to drive exocrine gland dysfunction.

The mechanisms responsible for higher susceptibility of female lacrimal glands to anti-Ro52 mediated dysfunction are not known. Inherent differences in gene expression between female and male glands might be responsible [35]. Regardless, considered together with our previous studies involving anti-Ro52 mediated salivary gland dysfunction, we would like to propose that antibody deposition within the exocrine tissues plays an important role in inducing glandular dysfunction. The experimental mouse model developed in this study provides an excellent tool to investigate the mechanisms responsible for the induction of dryness in Sjögren’s syndrome.

## 4. Materials and Methods

### 4.1. Proteins and High Titer Immune Sera

The production of purified recombinant MBP-mRo52 fusion protein and MBP was done as described before [25]. Polyclonal antibody service from Lampire Biological Laboratories (Pipersville, PA, USA) was used to obtain high titer rabbit anti-Ro52 and rabbit anti-MBP immune sera. Two rabbits were used per immunogen.

### 4.2. Mice

All the experiments described in this manuscript were approved by the Institutional Animal Care and Use Committee (Protocol Code#16-08, 24 May 2017). The NZM2758 mice were bred and maintained under specific pathogen-free conditions in the Oklahoma Medical Research Foundation vivarium. All mice had unrestricted access to food and water. All mice were maintained on a standard Teklad diet 7912.

Female and male mice were immunized either with MBP-mRo52 or MBP adsorbed on to adjuvant alum (ThermoFisher, Rockford, IL, USA), as described previously [25].

The tear production was measured using the Phenol Red Thread Test (Zone Quick; Menicon America INC, North Billerica, MA, USA), as described by Zoukhri et al. [6] Mice were anesthetized using Isoflurane, and a phenol red cotton thread was placed inside the lower eyelid for 30 s. The distance traveled by the tears (as indicated by the red coloration on the thread) was read immediately under a microscope equipped with a micrometer scale. Measurements were obtained from each eye and the results presented are an average of the readings from both eyes. Mice were bled at the time of euthanasia and serum stored at −80 °C till use.

### 4.3. Quantitative Immunoprecipitation Assay (IP)

The levels of serum anti-Ro52 antibodies were analyzed by a quantitative immunoprecipitation (IP) assay, as described previously [25]. Briefly, in vitro transcribed and translated ^35^S-Met-labeled mRo52 was generated and used as the substrate for the IP. IgG antibodies from mouse sera were captured onto Protein A sepharose beads (G-Biosciences, St. Louis, MO, USA), followed by incubation with ^35^S-Met-labeled mRo52. Bound radioactivity was measured by scintillation counting. A pool of mRo52 reactive sera was used as the positive control and antibody reactivity in sera from immunized mice was calculated as a percent of positive control reactivity.

### 4.4. Histopathology

Lacrimal glands were fixed in 10% buffered formalin and processed for paraffin embedding and sectioning using standard methods [25]. Sections (3-micron thickness) were stained with hematoxylin and eosin (H&E) and evaluated for the presence of inflammatory foci by an observer blinded to experimental design. The inflammation was scored on a scale of 0–4, with 0 indicating no inflammatory foci and 1–4 representing increasing severity.

### 4.5. Immunofluorescence Staining and Quantification of IgG Deposits in Lacrimal Glands

Formalin-fixed, paraffin-embedded lacrimal gland sections were deparaffinized and rehydrated in a decreasing alcohol gradient. Following heat-induced epitope retrieval performed under acidic conditions, the sections were incubated in Triton X-100 (0.1%) and normal horse serum to block nonspecific binding. Mouse IgG deposits were detected by incubation with goat anti-mouse IgG-Alexa Fluor 647 conjugate (Southern Biotech, Birmingham, AL, USA). Slides were then mounted with ProLong Diamond Antifade Mountant (ThermoFisher, Grand Island, NY, USA). Three to five non-overlapping areas of each stained lacrimal gland were captured using a 20x objective on a Zeiss Inverted Fluorescence Microscope. Using ImageJ software, a uniform threshold was set for all images and the area positive for IgG staining was quantified. Data are presented as percent IgG positive (IgG positive areas/total area × 100) for each lacrimal gland studied.

For mice treated with hyperimmune rabbit serum, lacrimal glands were fixed in paraformaldehyde-lysine-periodate, transferred to sucrose, and embedded in an optimum cutting temperature (OCT) compound for cryostat sectioning. Donkey anti-rabbit IgG-Alexa Fluor 647 conjugate (Abcam, Cambridge, MA, USA) was used for detection of rabbit IgG deposits using standard protocols [25].

### 4.6. Passive Transfer of Rabbit Serum into Adjuvant Treated Mice

Male and female mice were treated with alum adjuvant on days 0 and 10, as previously described [25]. On day 20, the mice were injected intraperitoneally with either anti-Ro52 or anti-MBP hyperimmune rabbit sera. The amount of serum injected in each mouse was normalized to deliver 15 mg/kg body weight of rabbit IgG. Tear production was measured after 24 h.

### 4.7. Statistical Methods

Graph Pad Prism software was used to perform all statistical tests. A normality test was performed on each data set and non-parametric tests (Mann Whitney and Kruskal Wallis with Dunn’s post-test) were used for non-Gaussian distributions. Parametric tests used for datasets following normal distributions include paired *t*-test, were used to compare changes in tear production before and after serum injection.

## Abbreviations

NZM	New Zealand Mixed
SSA	Sjögren’s Syndrome Antigen A
MBP	Maltose binding protein
IP	Immunoprecipitation
H&E	Hematoxylin and Eosin
